# Impact of China’s Rural Land Marketization on Ecological Environment Quality Based on Remote Sensing

**DOI:** 10.3390/ijerph191912619

**Published:** 2022-10-02

**Authors:** Zihao Li, Xihang Xie, Xinyue Yan, Tingting Bai, Dong Xu

**Affiliations:** 1 School of Business, Nanjing University of Information Science and Technology, Nanjing 210044, China; 2 Yangtze Institute for International Digital Trade Innovation and Development, Nanjing 210044, China; 3 School of Economics and Management, Northeast Electric Power University, Jilin City 132012, China; 4 School of International Business and Economics, Henan University of Economics and Law, Zhengzhou 450046, China; 5 School of Business Administration, Northeastern University, Shenyang 110189, China; 6 State Key Laboratory of Remote Sensing Science, Faculty of Geographical Science, Beijing Normal University, Beijing 100875, China

**Keywords:** market entry of rural collective operating construction land, rural land marketization, ecological environment quality

## Abstract

The market entry of rural collective operating construction land (MERCOCL) is an important way for the Chinese government to promote the marketization of rural land. However, the impact of China’s Rural Land Marketization on the ecological environment quality (EEQ) remains to be understood. Understanding these mechanisms is necessary for regional sustainable development and rational resource allocation. Therefore, a universal assessment model of China’s regional EEQ was built based on the Landsat 5/8 and the national ecological index (EI) provided by the Ministry of Ecology and Environment at the national district and county scale. A total of 229 counties (32 pilot counties and other counties in the pilot cities) in China from 2011 to 2018 were taken as the research object. This paper empirically studied the evolution process, driving mechanism and spatial heterogeneity of EEQ from the perspective of MERCOCL. The study shows that China’s EEQ presented a spatial distribution pattern of “high in the south, low in the north, high in the east and low in the west”. When a county implemented the MERCOCL policy, its EEQ index increased by 0.342, with the improvement effect occurring in the second year after the MERCOCL implementation. Regarding the mechanism, MERCOCL mainly improved the EEQ by promoting industrial structure optimization and increasing urban population aggregation. From the perspective of spatial heterogeneity, the improvement effect of MERCOCL on EEQ was more significant in regions with lower economic development levels and latitudes (southern China). This study will facilitate an understanding of the impact of China’s rural land marketization on the EEQ and provide scientific data support for government departments to formulate sustainable urban development policies that meet local conditions.

## 1. Introduction

China’s dual land ownership system that divides urban and rural areas has allowed local governments to monopolize land supply in the primary land market. Local governments can acquire agricultural land for large-scale development and transfer it at lower prices. Then, local governments use the low transfer price of industrial land as an effective means to attract an inflow of investment. In addition, local governments provide commercial and residential land at higher prices to compensate for the lack of local government financial resources by obtaining high land transfer revenue. This double second-hand land supply strategy fully utilizes the land’s economic value and facilitates China’s regional economy [1]. However, it has also significantly damaged the regional ecological environment (EEQ) through pathways such as strengthening extensive economic growth, inhibiting industrial structure upgrading and hindering technological innovation [2]. According to *the Chinese Ecology and Environment Status Bulletin*, 53.4% of 337 Chinese cities did not meet the annual air quality standard in 2019. Numerous studies have shown that China’s long-term non-market-based land transfer has caused unreasonable industrial land planning, inefficient land use and backward industrial structure. Thus, this has a direct negative impact on regional air pollution [2,3,4].

In 2015, the Chinese central government issued the *Opinions on the Pilot Work of Rural Land Acquisition*. The market entry of rural collective operating construction land (MERCOCL) and the Homestead System reform were introduced into pilot implementation. Particularly, the MMERCOCL reform has broken the monopoly of the pilot local government on the primary land market and thus allowed the price mechanism to allocate land resources. It is regarded as an important measure for the Chinese government to promote rural land marketization and for the Chinese central government to reduce land market distortions, improve land use efficiency and upgrade regional industrial structure [5,6,7]. Research in related fields shows that with the reduction in regional land market distortion, regional pollution-intensive industry entry [8] and air pollution [2] are significantly reduced. With the improvement of regional land use efficiency and industrial structure transformation and upgrading, regional energy conservation, emission reduction and air pollution control are effectively improved [2,9]. Therefore, as an important part of the rural land marketization reform, MERCOCL also impacts regional environmental quality.

In this paper, MERCOCL was used as an important indicator to measure China’s rural land marketization. Then, the ecological environmental effects of China’s rural land marketization were analyzed. Based on the Landsat 5/8 satellite and national ecological index (EI), a general regional EEQ assessment model in China was constructed to objectively evaluate the EEQ of 32 experimental areas and their neighboring counties. This paper addresses the following major issues: (1) What are the spatio-temporal differences in EEQ in the reform pilot areas? (2) Will MERCOCL improve China’s EEQ? (3) If MERCOCL positively impacts EEQ, what is the driving mechanism for MERCOCL to improve EEQ? (4) Considering the complexity of MERCOCL, is there any spatial heterogeneity in the impact of MERCOCL on EEQ? This study is of great significance in China’s efforts to promote market-oriented allocation of land factors and continuous pollution control.

This paper is organized as follows: following the Introduction, Section 2 is the literature review; Section 3 is the theoretical mechanism analysis; Section 4 presents the measurement method and data sources; Section 5 demonstrates the research results; Section 6 presents the conclusions.

## 2. Literature Review

Many studies have been focused on the MMERCOCL in China. Firstly, many scholars have focused on the necessity and importance of the MMERCOCL reform. For example, Guo et al. (2015) regarded the reform as an important means to promote land marketization in China [10]. It effectively broke the monopoly of local governments on land management rights and promoted the equality and integration of urban and rural construction land rights [11]. Gao et al. (2020) took it as an important strategic measure of rural industrial transformation and upgrading. Secondly [5], some scholars have focused on the barriers broken by MERCOCL. For example, through a principal–agent model, Yan et al. (2021) found that a reasonable and optimized benefit distribution mechanism was an important guarantee for promoting the reform [12]. Wang (2022) analyzed the interview data from five pilot regions and showed that effective coordination of the short-term interests of farmers and the long-term interests of rural collectives was crucial to the reform [13]. Finally, with the further promotion of MERCOCL, an increasing number of scholars have started to show their concerns about the effects of MERCOCL. For example, Wen et al. (2022) found that MERCOCL effectively alleviated local construction land shortages by analyzing the pilot projects in Deqing County of Zhejiang Province and Nanhai District of Guangdong Province [14]. Tian et al. (2020) studied the reform in four first-tier cities in China [15]. Wen et al. (2020) explored the MERCOCL reform in the Nanhai District of Guangdong Province and found that the MERCOCL reform was ineffective in reducing local house and land prices due to local government revenue pressure [16]. Based on a case study in Deqing, Zhejiang Province, Wang and Tan (2020) found that it effectively improved the local land allocation efficiency and directly raised farmers’ income levels [17]. Various studies on MERCOCL have focused on status-quo discussions and case-based empirical analysis. Large-sample quantitative empirical studies have been rarely conducted. In addition, existing studies on the impact of the reform are still limited to conventional aspects such as land and income. However, few studies have examined the impact of MERCOCL on regional ecology.

In terms of the impact of land use on EEQ, existing studies have been carried out from three aspects. Firstly, many scholars have used the comprehensive econometric regression model and the land-use regression model proposed using the GIS technology of Briggs et al. (1997) to predict and analyze the distribution of environmental pollution at the urban scale [18]. For example, previous studies on the distribution of urban environmental pollution show that the planning of urban land use has an important influence on environmental pollution [19,20,21,22]. Secondly, the effects of different land use types on EEQ have been studied. For example, Lu et al. (2020) found that artificial surfaces, arable land and desert could deteriorate EEQ, while forests and grasslands could effectively improve EEQ [23]. This is consistent with the studies by Lin et al. (2020) and Xu et al. (2021) [24,25]. Thirdly, the influence of urban land-use efficiency on environmental pollution has been studied. Li et al. (2021) found an inverted U-shaped relationship between industrial land-use efficiency and environmental pollution using data from 270 Chinese cities from 2015 to 2018 [4]. Liu et al. (2021) found that the mismatch of land resources exacerbated environmental pollution in local and neighboring regions by hindering industrial structure upgrading and inhibiting technological innovation [2]. However, the effect of land reform policies on environmental quality in China has been rarely investigated. Finally, some scholars have also explored the impact of China’s rural land marketization on EEQ by taking different reform policies as examples. For example, based on an empirical study of 140 villages in five Chinese provinces, Xu et al. (2018) found that the Three Rights Separation Policy enabled farmers to increase the organic fertilizer input and promoted the agricultural ecological environment [26]. Chen (2021) took an urban area in Yan’an, Shaanxi, China, as an example and found that rural residential land expansion deteriorated the ecological environment around the town [27]. Yang (2018) believed that rural land circulation would significantly impact the rural ecological environment and constructed a more systematic rural land circulation environmental performance evaluation index system [28]. In addition, few studies have used China’s high-precision and high-resolution EEQ data to investigate the impact of land use policies on EEQ from a micro perspective. Studies based on the MERCOCL reform are even rarer.

Despite the current research progress, there are still some shortcomings. Firstly, based on statistical or remote sensing data, previous studies lack an accurate evaluation of EEQ data. Secondly, existing studies on the impact of MERCOCL reform are still limited to conventional aspects such as land and income. Few studies have examined the impact of MERCOCL on regional ecology. Thirdly, although existing MERCOCL studies are abundant, they are mostly status quo discussions or case-based empirical analyses. Quantitative empirical studies with large samples are relatively rare. Therefore, based on the panel data of 229 Chinese counties (including 32 pilot counties) from 2011 to 2018, the spatial and temporal patterns of EEQ based on remote sensing data were analyzed. Furthermore, the impacts of MERCOCL on EEQ, the driving mechanism and spatial heterogeneity were also empirically investigated using the difference-in-differences (DID), the propensity score matching (PSM) model and the intermediary effect method.

The contributions are as follows. Firstly, this article used Landsat images nationwide and the national county EI index data provided by the Ministry of Ecology and Environment to construct a high-resolution and high-precision assessment model of China’s EEQ. This model showed good universality and robustness. Based on this model, China’s EEQ data sets from 2011 to 2018 were produced. The distribution pattern and evolution process of EEQ in pilot cities in the recent ten years were also discussed. Secondly, MERCOCL was regarded as a quasi-natural experiment for the first time to investigate the impact of rural land marketization on EEQ. This study extends the literature on land use and EEQ. Thirdly, from the perspective of local industrial structure and population aggregation, this study examined the driving mechanism of the impact of the rural land market on EEQ. The impact mechanism of MERCOCL on EEQ was discussed. This will provide a valuable reference for improving the EEQ in subsequent pilot regions.

## 3. Theoretical Mechanisms

In 2015, MERCOCL enabled rural land marketization; in 2020, the newly revised China Land Administration Law confirmed the MERCOCL nationwide legal status. The law clearly stated that eligible collective operating construction land could be reasonably transferred to entities and individuals for direct use, eliminating important institutional obstacles in China’s rural land marketization.

### 3.1. Impact Direction of the MERCOCL on EEQ

China’s original urban-rural dual land system induces ambiguous rural land ownership rights. To obtain sufficient and sustained capital for economic development, local governments expropriate agricultural land at low prices [12], sell industrial land cheaply by reaching an agreement and even reduce the investment quality by attracting enterprises with backward production capacity and high pollution [29]. Thus, this will impair EEQ. MERCOCL reported that urban and rural construction land should be equally entered into the market and the principle of the same rights and price should be strictly observed. This will reduce the EEQ deterioration caused by land resource misallocation [2]. Then, MERCOCL is conducive to forming construction land prices and land marketization mechanisms that reflect the market demand. The effective operation of the land market mechanism will further improve the efficiency of urban construction land. In turn, EEQ through improved energy efficiency and industrial structure upgrading can be improved [4,9]. In addition, MERCOCL can actively enable the market to allocate land resources by bypassing land acquisition. This is conducive to reducing the local governments’ environmental competition to the bottom line due to land financing [30]. Based on the above analysis, the following hypothesis was proposed in this study:

**Hypothesis** **1.** *MERCOCL can improve EEQ.*

### 3.2. Driving Mechanism of the MERCOCL on EEQ

#### 3.2.1. Industrial Structure Optimization and EEQ

Firstly, China’s industry-dominated secondary industry still has high energy consumption, which hinders the adjustment and upgrading of green industries and the improvement of EEQ [31]. Secondly, the discriminatory land supply strategy led by local governments can inhibit the development of China’s service industry, lead to the rigidity of the industrial structure, hinder the high-end upgrading of the manufacturing industry, allow the development of local middle and low-end manufacturing industry and worsen EEQ [8]. Finally, seeking economic development based on land use can distort land resource allocation [2]. Land resource misallocation can attract investment by increasing the scale of industrial land transfer and lowering the industrial land price. This may result in various issues. Over-industrialization of industrial structures causes increased energy consumption and industrial pollutants, thus exacerbating EEQ deterioration [2]. This indicates that rational land resource allocation can improve EEQ by optimizing the industrial structure. MERCOCL can increase the urban operating construction land supply, facilitating the development of reasonable land prices. This can reduce the phenomenon that the government attracts backward industries to enter at a significantly low price and reduce environmental pollution due to excessive industrialization. Thus, this hypothesis was proposed:

**Hypothesis** **2.1.** *MERCOCL can improve EEQ by optimizing industrial structure.*

#### 3.2.2. Government Intervention Reduction and EEQ

As the main supplier of public goods, the government bears the responsibility of environmental protection and governance. However, when the central government’s environmental protection constraints on local governments are not enforced, local governments can actively intervene in the land market transfer orientation and the actual enforcement standards of regional environmental regulations in order to achieve short-term economic growth. Local governments would introduce backward enterprises at the expense of the environment, thus reducing the provision of quality environmental public goods and worsening EEQ [32]. Local government intervention in land resource allocation is mainly due to their monopoly on resources in the land market and excessive intervention in the land transaction market. Such practice is detrimental to technological progress in energy conservation and emission reduction [9], thereby hindering the improvement of local EEQ. However, implementing the MERCOCL pilot has promoted land marketization, which has weakened local governments’ monopoly on the land market to some extent [11]. Along with the reform of the regulation of local government’s land acquisition practices, the restraint on local government’s intervention in land transfer can be significantly enhanced, which is beneficial to the EEQ improvement. Therefore, the following research hypothesis was proposed:

**Hypothesis** **2.2.** *MERCOCL can improve EEQ by reducing excessive governmental intervention.*

#### 3.2.3. Urban Population Agglomeration and EEQ

The impact of population agglomeration on EEQ mainly depends on whether the population agglomeration has a scale effect or an intensive effect. When the population density is lower, the infrastructure construction in towns and cities is underdeveloped and the investment in public services is seriously insufficient. In order to obtain sufficient funds to support urbanization construction, local governments would attract investment through unreasonable land transfer. This can increase pollution sources [33] and induce population agglomeration’s scale effect. When the population scale further expands, the public infrastructure in cities is improved, with more convenient transportation. Therefore, the amount of environmental pollution handled by local governments will exceed the number of emissions, thus causing an intensive effect of population agglomeration [34]. Urban housing prices are inflated [16] and there is a crowding-out effect on urban population inflow to some degree, which is not conducive to the intensive effect of population agglomeration. However, MERCOCL can promote urban-rural integration and regulates land development during urbanization. Generally, excessive real estate prices can be curtailed, which will help attract people to cities. Then, government investment in environmental pollution governance can increase accordingly [33]. Population agglomeration can share the positive spillover effects of public infrastructure and reduce energy and transportation costs. The unit cost of shared pollution treatment facilities is lower than that of separate pollution facilities. The scale effect of pollution treatment can be fully utilized to help improve EEQ [35,36]. Therefore, implementing the MERCOCL policy may stimulate intensive population aggregation and make the intensive effect more significant than the scale effect, thus improving EEQ. Based on the above analysis, the following research hypothesis was proposed:

**Hypothesis** **2.3.** *MERCOCL can improve EEQ through the intensive effect of population agglomeration.*

## 4. Data Sources and Methodology

### 4.1. Data Description and Sources

#### 4.1.1. Explained Variable

The explained variable was the ecological environmental quality (EEQ). In econometrics, the dependent variable can also be called the explained variable. EEQ is both the dependent variable and the explained variable in this study. By referring to the practice of Xu et al. (2021b) [37], a universal assessment model of China’s regional EEQ was built based on the Landsat 5/8 surface reflectance and the national EI provided by the Ministry of Ecology and Environment at the national district and county scale. All Landsat surface reflectance data in GEE were de-clouded and stripped. Then, all remote sensing data were resampled to 1000 m using the nearest neighbor interpolation. For a detailed description of the remote sensing data used used in this study, please refer to Table 1.

#### 4.1.2. Explanatory Variables

In February 2015, the Standing Committee of the National People’s Congress passed the Decision on Authorizing the State Council to Temporarily Adjust the Implementation of Relevant Laws and Regulations in the Administrative Areas of 33 Pilot Areas regarding the relevant provisions of rural land acquisition, MERCOCL and homestead management system. The pilots for these three reforms were proposed and a task was arranged for each pilot area, i.e., a pilot area implements one pilot task. The 33 pilot areas consisted of 15 for MERCOCL, 15 for the homestead system reform and 3 for the land acquisition system reform (due to missing data, Qushui County in Tibet Autonomous Region was excluded). For the homestead reform, the pilot task mainly included (1) exploring the implementation of paid use of homesteads that exceed the standard due to historical reasons; (2) exploring the voluntary withdrawal or transfer of homesteads within the collective economic organization by farmers who have settled in cities; (3) reforming the homestead approval system and promoting the democratic management role of villagers’ self-governing organizations. In terms of MERCOCL, the regulation states that on the premise of compliance with planning, use control and legal acquisition, the stock of the rural collective operating construction land use rights is allowed to be transferred, leased and invested in shares. These measures are significant to China’s rural land marketization reform.

**Figure 1:**
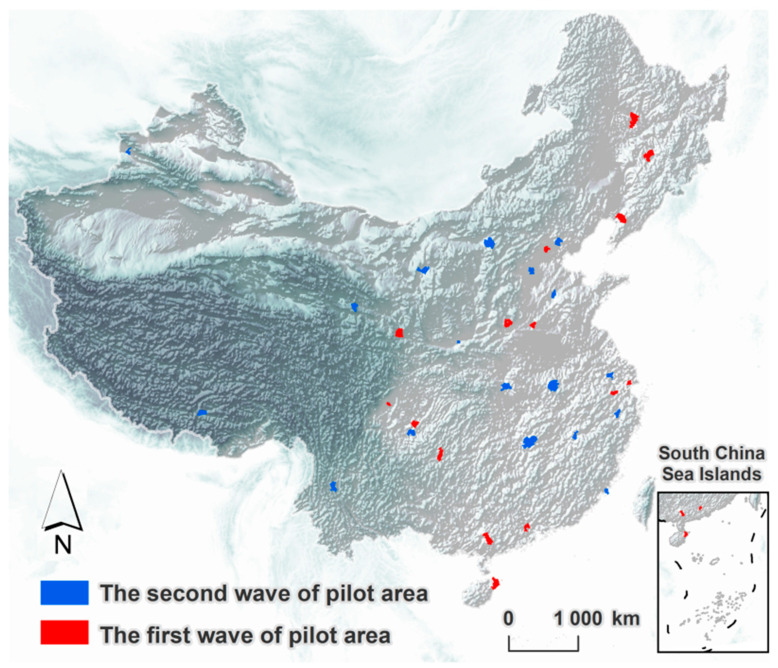
Geographical distribution of pilot areas.

In September 2016, the Central Leading Group for Comprehensively Deepening Reform decided to expand the MERCOCL and land acquisition system reform to all 33 pilot areas. Furthermore, to better reflect the integrity, systematic and synergistic nature of the rural land system reform, the pilot period was extended to 31 December 2018. In the pilot promotion process, the central government considered the regional environment, resource endowment and economic development level to achieve a comprehensive provincial-wide rollout from coastal to inland. This also reflected the strategic planning by expanding out from one point. Figure 1 shows the geographical distribution of the 33 pilot areas.

The dummy variables of the MERCOCL policy were used as explanatory variables, i.e., *dt_it_*: “1” indicates that the county *i* launches the MERCOCL in *t* year; otherwise, the value is 0. 

#### 4.1.3. Control Variables

To reduce the bias of other potential factors on the estimated results, control variables *X* were added. Economic growth (ey)and its squared term (ey^2^) were expressed by actual GDP per capita and the squared term of actual GDP per capita, respectively. These two variables were used to test whether there was an Environmental Kuznets Curve (EKC) in this study [38]. The industrial structure (s) was expressed as the proportion of the added value of the secondary industry in each region’s GDP. The population density (den) was measured by the ratio of the total population to the total area of the region. The natural flow of the atmosphere caused by weather factors (such as wind speed, temperature difference and rainfall) can also significantly impact local EEQ. The annual average temperature (temp) and annual total rainfall (rain) of counties were introduced as the control variables of meteorological factors.

#### 4.1.4. Intermediary Variables

Intermediary variables: industrial structure optimization (str) was expressed as the proportion of the added value of the tertiary industry to regional GDP. Government intervention (gov) was measured by government fiscal expenditure as a percentage of regional GDP. Urban population agglomeration (pop) was expressed as the ratio of the non-agricultural population to the total population.

The EI index provided by the Ministry of Ecology and Environment of China ended in 2018. The MERCOCL policy was first implemented in 2015. Thus, the first four and last four years were selected as the research period (including the year in which the policy was implemented). Therefore, 229 counties in the cities where 32 pilot counties are located from 2011 to 2018 were selected as the research objects. The indicators involving price factors in the data were treated at constant prices using the fixed price index in 2000 as the base period. The data used in this paper were obtained from China National Meteorological Science Data Center, the Ministry of Ecology and Environment, *the China City Statistical Yearbook*, *Provincial Statistical Yearbooks* and the official websites of provincial finance departments. Table 2 shows the indicator definition and descriptive statistics of the variables. 

### 4.2. Methodology

#### 4.2.1. Evaluation of EEQ

The original remote sensing ecological index (RSEI) model [39] incorporated remote sensing-based greenness, humidity, heat and dryness to assess regional eco-environmental quality. Based on this model, this study developed a new EEQ evaluation model for remote sensing data and produced China’s high-resolution ecological environmental quality data (*CHEQ*).

(1) $$CHEQ=\frac{PC1-PC1_{\min}}{PC1_{\max}-PC1_{\min}}$$

(2) PC1=PCA(NDVI,NDBSI,LST,WET,AI)

where *CHEQ* was the EEQ index, *PC*1 was the first principal component, *PC*1_min_ and *PC*1_max_ were the minimum and maximum values of *PC*1, and *NDVI*, *NDBSI*, *LST*, *WET*, and *AI* were greenness, dryness, heat, humidity and abundance index for land cover type, respectively [39].

The traditional RSEI model was often used to monitor the EEQ at the local scale. Additionally, a few scholars [40] adopted the RSEI model to evaluate China’s EEQ, but none considered the RSEI model’s applicability in China. Therefore, we improved RSEI and then evaluated the applicability of the RSEI and *CHEQ* in China.

In particular, the EI calculation greatly depended on the abundance index of land cover types (*AI*). In contrast, four of the aforementioned indexes in the RSEI model did not involve the important evaluation factor. Therefore, based on the “Technical Criterion for Ecosystem Status Evaluation” [41], the *AI* in the study area during 2011–2018 was calculated based on the MCD12Q1 data:

(3) $$\begin{array}{ll} AI & =\mu \times (0.35\times Forest+0.21\times Grassland+0.28\times Water+\\ & 0.11\times Cropland+0.04\times Built+0.01\times Unused)/Area \end{array}$$

where *AI* was the abundance index for land cover types; *μ* was the normalized coefficient; *Forest*, *Grassland*, *Water*, *Cropland*, *Built* and *Unused* were the area of forest land, grassland, waterbody, cropland, built-up and unused land, respectively; *Area* was the total area.

#### 4.2.2. Econometric Model Setting

The exogenous event of MERCOCL pilot reform was regarded as a quasi-natural experiment. In this study, 15 counties in the first batch and 17 counties in the second batch (Qushui County in Tibet Autonomous Region was excluded due to missing data) were taken as the pilot policy research objects. The DID method was used to explore the impact of MERCOCL on EEQ. Specifically, the 32 pilot counties of MERCOCL were considered as the treatment group. The other 197 non-pilot counties in the prefecture-level cities of 32 pilot counties were taken as the control group.

The econometric estimation was conducted from the following three aspects:

(1) To empirically analyze the impact of MERCOCL on regional EEQ, this paper examined the differences in EEQ between pilot and non-pilot counties before and after the implementation of the MERCOCL policy. Since the pilot counties launched the MERCOCL system in 2015 and 2016, respectively, the DID model for consecutive years was used

(4) $$EEQ_{it}=\alpha_{0}+\alpha_{1}dt_{i}{}_{t}+\gamma Χ+\lambda_{i}+\lambda_{t}+\mu_{it}$$

(2) Due to the differences in income level of residents and resource endowment between different regions, the econometric estimation results are affected. Errors were eliminated by matching the treatment and control groups using PSM and then the endogeneity problem was solved through DID analysis. Therefore, a combined method (PSM-DID) was used to conduct robustness tests.

(5) $$EEQ_{it}{}^{psm}=\alpha_{0}+\alpha_{1}dt_{i}{}_{t}+\gamma Χ+\lambda_{i}+\lambda_{t}+\mu_{it}$$

where *i* is county level, *t* is the year, EEQ is the ecological environmental quality and *dt* is the policy dummy variable representing whether the area belongs to the treatment group. The counties where MERCOCL was initiated were taken as the treatment group and *dt* = 1; the other 197 counties were taken as the control group and *dt* = 0. The coefficient $\alpha_{1}$ was the core parameter; $\alpha_{1}>0$ indicates a positive effect of MERCOCL on EEQ, i.e., MERCOCL can improve EEQ; $\alpha_{1}<0$ indicates a negative effect of MERCOCL on EEQ, i.e., MERCOCL will worsen EEQ. In addition, a series of control variables X were added to Equations (4) and (5) to avoid the deviation of measurement results caused by omitted variables.

(3) If MERCOCL can improve EEQ, by what pathway does it affect EEQ? The mechanism analysis suggests that MERCOCL may improve EEQ through industrial structure optimization and reduce government intervention and population aggregation. This study referred to the three-step test of Baron and Kenny to develop an estimation model, thus verifying the existence of the driving mechanism [42]. The first step is to verify whether the MERCOCL can promote industrial structure optimization, reduction in government intervention and population aggregation. The second step is to verify whether the MERCOCL can affect EEQ. In the third step, *str*, *gov* and *pop* were added to the equation in the second step for regression. If the coefficient of *dt* in Equation (8) is insignificant or significant but smaller, this indicates that MERCOCL can improve EEQ through these three pathways.

Step 1: Verify the impact of MERCOCL through the three pathways.

(6) $$str_{it}(gov_{it},pop_{it})=\chi_{0}+\chi_{1}dt_{i}{}_{t}+\phi X+\lambda_{i}+\lambda_{t}+\pi_{it}$$

Step 2: Verify the impact of MERCOCL on EEQ.

(7) $$EEQ_{it}=\alpha_{0}+\alpha_{1}dt_{i}{}_{t}+\gamma X+\lambda_{i}+\lambda_{t}+\mu_{it}$$

Step 3: Add *str*, *gov* and *pop* to Equation (5).

(8) $$EEQ_{it}=\delta_{0}+\delta_{1}dt_{i}{}_{t}+\theta str_{it}(gov_{it},pop_{it})+\varphi X+\lambda_{i}+\lambda_{t}+{ο}_{it}$$

where *str* represents industrial structure optimization; *gov* represents government intervention; *pop* represents population agglomeration; $\chi_{0}$ and $\delta_{0}$ are the intercept terms; $\chi_{1}$ and $\delta_{1}$ are the core explanatory variable coefficients; θ represents the influence coefficients of *str*, *gov* and *pop*; ϕ and φ are the control variable coefficients; π and ο are the random perturbation term.

## 5. Results and Discussion

### 5.1. The Verification of EEQ

**Figure 2:**
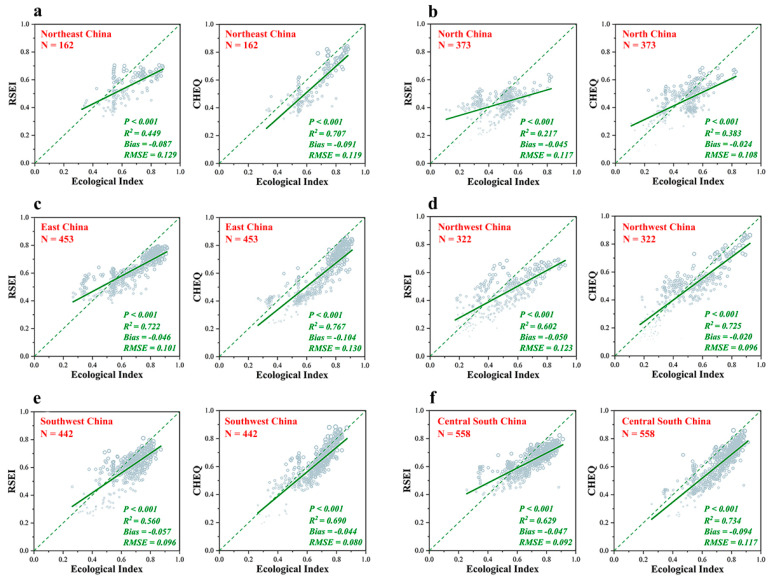
Accuracy verification of CHEQ and RSEI based on ecological index (EI) provided by the Ministry of Ecology and Environment of the People’s Republic of China (MEEPRC): (**a**) Accuracy verification of CHEQ and RSEI in northeast China; (**b**) Accuracy verification of CHEQ and RSEI in north China; (**c**) Accuracy verification of CHEQ and RSEI in east China; (**d**) Accuracy verification of CHEQ and RSEI in northwest China; (**e**) Accuracy verification of CHEQ and RSEI in southeast China; (**f**) Accuracy verification of CHEQ and RSEI in central south China.

Figure 2 shows the accuracy verification comparison between the improved EEQ evaluation model and the RSEI model. Considering China’s vast geographical area and complex and diverse land cover types, the two models were validated according to different geographical regions (northeast, north, east, northwest, southwest and central south). From Figure 2, CHEQ showed superior accuracy compared with the RSEI model in different partitions. In all partitions, the fitting degree of CHEQ and EI data was better than that of RSEI. The fitting degree in the eastern region was the highest (0.707), followed by the central south region (0.734), the northwest region (0.725), the northeast region (0.707) and the southwest region (0.690). The fitting degree in the northern region was the lowest (0.383). In terms of root mean square error (RMSE), the RMSE of CHEQ was better than that of the RSEI model in four regions, i.e., northeast (0.119), north (0.108), northwest (0.096) and southwest (−0.080). In terms of BIAS, CHEQ’s BIAS was better than RSEI’s in the northern region (−0.024), northwest region (−0.020) and southwest region (−0.044).

### 5.2. Temporal and Spatial Variation of EEQ

**Figure 3:**
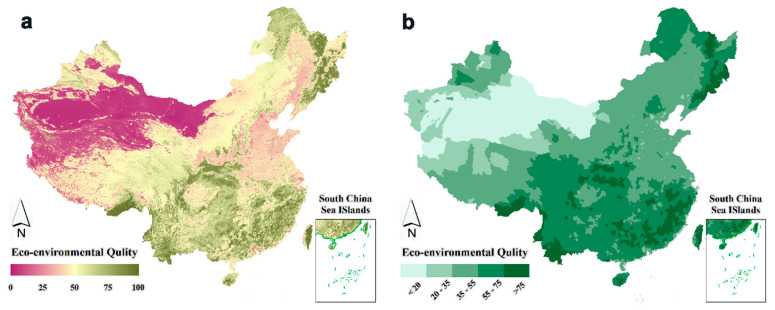
Spatial distribution maps of EEQ (CHEQ) in China in 2018. Spatial distribution map of EEQ in China in 2018 (**a**) at the 1000 m grid scale and (**b**) at the country scale.

Figure 3 shows the spatial distribution map of China’s EEQ in 2018. It can be seen that the spatial distribution of China’s EEQ presented a pattern of “high in the south, low in the north, high in the east and low in the west”. Furthermore, in areas with densely distributed forests, the EEQ was generally higher. For example, the EEQ of China’s three major forest areas (Northeast Forest Area, Southwest Forest Area and Southern Forest Area) was almost 100. This is because dense forests can provide high water and soil conservation functions, sand and wind-fixation capabilities and higher biodiversity. On the contrary, the sparse vegetation in the northwest desert region, perennial drought and high temperature made this region’s ecological environment significantly harsher [43].

**Figure 4:**
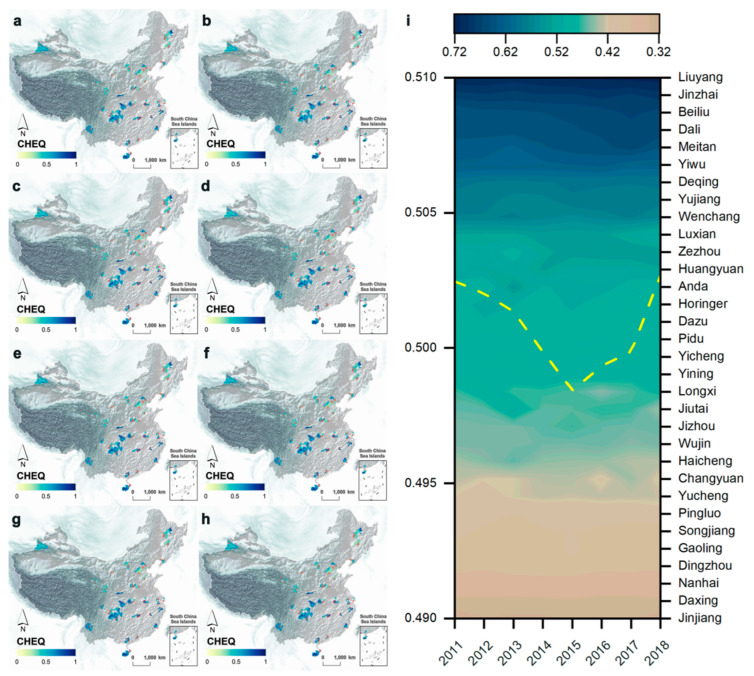
Spatial and temporal variation maps of EEQ in 229 counties: (**a–h**) Spatial distribution maps of EEQ in 229 counties from 2011 to 2018; (**b**) EEQ heat map of 32 pilot counties from 2011 to 2018 (**i**).

From Figure 4, the spatial distribution of EEQ in 229 counties showed a pattern of “high in the south, low in the north, high in the east and low in the west” (Figure 4a–h). Secondly, in the 32 pilot counties, Liuyang City had the highest EEQ in the past ten years, with an average of 0.714, followed by Jinzhai County (0.682), Beiliu City (0.656), Mardan County (0.683) and Meitan County (0.639). However, Jinjiang City had the lowest EEQ (0.324), followed by Daxing (0.330), Nanhai (0.359), Dingzhou (0.382) and Gaoling (0.386). In addition, from Figure 3, the EEQ of the 32 pilot counties experienced a U-shaped change of declining and then rising in the past ten years. The inflection point was in 2015 with an average value of (0.498), indicating that the U-shaped changes in EEQ of pilot counties were likely to be influenced by MERCOCL policies.

### 5.3. Direction Analysis of the Impact of the MERCOCL on EEQ

Based on remote sensing big data, the spatio-temporal evolution pattern of EEQ in 229 counties, including 32 pilot counties, was analyzed. It was found that 32 pilot counties showed significant improvement after the implementation of the MERCOCL policy. Therefore, this paper used econometric methods to explore whether EEQ was affected by MERCOCL.

In this section, the impact of MERCOCL on EEQ was empirically examined using the DID method. To avoid multi-collinearity, a stepwise regression method was adopted, i.e., control variables were gradually added into the model to examine the effects of MERCOCL on EEQ. In addition, since the variables studied were all concentrated at the county level, the robust standard error was used for regression analysis. The specific results are shown in Table 3.

In Table 3, Column (1) is the measurement result only including policy dummy variables *dt*. The coefficient of *dt* was 0.342 and passed the significance test, indicating that MERCOCL can significantly improve EEQ. When a county implemented the MERCOCL policy, its EEQ index increased by 0.342. The estimation results verified the existence of Hypothesis 1. Compared with existing studies on the impact of land resource misallocation (non-marketization of land transfer) on the environment [2,30], this study verified for the first time that MERCOCL, a land marketization policy, can improve EEQ. This is because improved land marketization can optimize urban construction land layout, improve land resource use efficiency and reduce environmental pollution. Therefore, the MERCOCL policy, as an important link in promoting land resource marketization, had significantly improved EEQ. Columns (2)–(7) are the inclusions of adding the control variables. After the above control variables were gradually added, the econometric estimated coefficient of *dt* was still significantly positive, indicating that the MERCOCL can improve EEQ. In other words, MERCOCL did improve EEQ. Although MERCOCL may be affected by other factors, it still had a promoting effect on EEQ.

As to other explanatory variables, the overall effect of economic growth (*ey*) on EEQ was significantly positive, while *ey*^2^ was significantly negative. At a relatively lower level of economic development, economic growth can improve EEQ. However, as the economic growth level increased, the corresponding energy consumption and pollution emissions increased, worsening EEQ. This is consistent with the conclusion of Song (2021) [44]. The coefficient of *s* was significantly positive. This conclusion is rather puzzling and consistent with the research conclusions of [4]. In recent years, China’s economic restructuring has made some progress. Its industrialization has gradually transformed into green development, thus helping to improve the quality of the ecological environment. The coefficient of (*den*) was negative but insignificant, showing that urban population density has no obvious effect on EEQ. This conclusion is similar to that of [45]. This is because the increase in population density leads to increased artificial land use. Then, the scale economy effect of local government environmental governance becomes more significant [34]. The coefficient of the annual total rainfall (*rain*) was significantly positive, which is similar to the conclusion of [23]. Rainfall can help improve the quality of the environment by reducing pollutants in the atmosphere. The coefficient of *temp* was negative but insignificant, indicating no obvious relationship between temperature and EEQ in the pilot counties. This research conclusion is similar to that of [46], and there is no fixed and necessary relationship between climate temperature and regional pollution.

To avoid bias due to differences in the changing trends of the pilot and non-pilot counties, the PSM was first used to match the propensity scores of the MERCOCL pilot and non-pilot counties. The Kernel matching method was used to calculate an estimated effect of all samples of the treatment group and control group to match the two groups. Then, DID was applied to the matched counties. Before performing PSM, the common support hypothesis was tested. The test results are shown in Table 4. It can be found that there was no significant difference between variables, which meets the premise of PSM-DID estimation.

Table 5 shows the results of the PSM-DID method. The coefficient of MERCOCL (*dt*) on EEQ was still significantly positive. There was no significant difference between PSM-DID and DID. EROCLM can significantly improve EEQ, further verifying the previous estimation’s robustness.

### 5.4. Driving Mechanism Analysis of the Impact of the MERCOCL on EEQ

Table 6 shows the driving mechanism test results of MERCOCL on EEQ. Columns (2) and (6) show that MERCOCL (*dt*) significantly promoted industrial structure optimization and urban population agglomeration. However, the results in Column (4) show that MERCOCL had no obvious effect on government intervention. Column (1) shows that MERCOCL had a significant improvement effect on EEQ. In the third step, industrial structure optimization (*str*), urban population agglomeration (*pop*) and government intervention (*gov*) were added to the base model. Columns (3) and (7) show that EROCLM can promote structure optimization and increase urban population agglomeration to improve EEQ in the pilot counties. This supports the conclusions of existing studies. For example, land marketization can reduce regional environmental pollution by optimizing regional industrial structure [2]. The efficient urban population agglomeration also benefited environmental governance [34]. However, Column (5) shows that MERCOCL had an insignificant effect on improving EEQ by reducing government intervention. The results show that the land marketization due to the MERCOCL policy provided more land use resources for industrial development and formed a more reasonable land transfer price [30]. Thus, these solved the problem of limited construction land and prevented the government from attracting backward industries that could bring rapid profits at a low price. However, due to the uncertainty of the impact of government intervention on environmental quality, the effect of land marketization on EEQ through government intervention did not exist [47]. Therefore, these conclusions proved the existence of Hypotheses 2.1 and 2.3 except for Hypothesis 2.2.

### 5.5. Parallel Trend Test and Robustness Test

**Figure 5:**
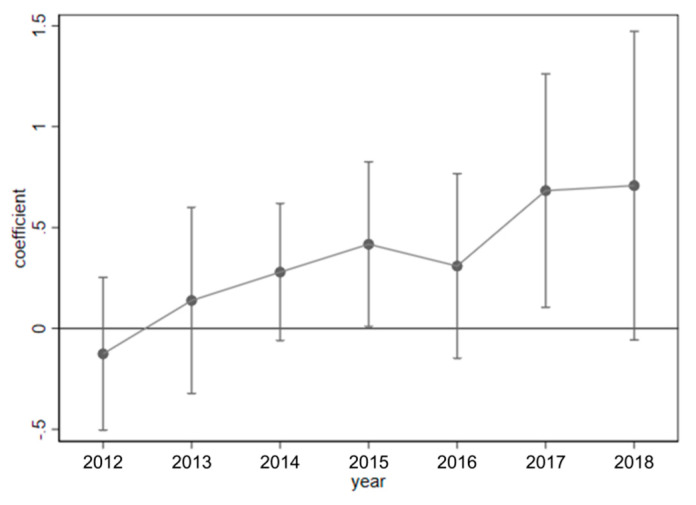
Parallel trend test.

When the DID method is used for econometric estimation, an important premise needs to be satisfied: without exogenous policy shocks of MERCOCL, the development trend of EEQ in pilot and non-pilot counties should be the same and not significantly different. In order to avoid multicollinearity, the base period was removed during the parallel trend test. The result is shown in Figure 5.

Figure 5 shows that before the MERCOCL policy implementation, the policy’s impact coefficients on EEQ were all around 0 and insignificant, indicating no significant difference in the changing trend between the pilot and non-pilot counties. This met the assumption of a parallel trend. In addition, after all regions joined the pilot cities, the improvement effect of the MERCOCL policy on EEQ did not occur until the second year after the policy implementation. This indicates the possible difficulties in the actual implementation of the reform. Tian (2020) also found that in the early stage of the MERCOCL, local governments resisted maintaining land fiscal revenue, thus hindering the smooth implementation of the policy [15]. Thus, there is a certain time lag from the beginning of the pilot to the actual implementation. In addition, it will take some time for MERCOCL to play its ecological value. 

In addition, to investigate whether the impact of MERCOCL on EEQ is affected by changes over sample time, the robustness of the MERCOCL policy on EEQ was tested by analyzing different periods. Table 7 shows that EROCLM significantly improved EEQ, which further verified the robustness of the results. 

The impact of MERCOCL on EEQ can inevitably be interfered with by other policies, leading to overestimation or underestimation of the effect of the MERCOCL. To identify and solve this problem, this paper obtained other relevant policy events that occurred before and after the MERCOCL implementation. In addition, the former Ministry of Environmental Protection put forward the environmental protection interview system in 2014 mainly for local governments with excessive emissions of key pollutants and carbon dioxide and urged them to actively fulfill their environmental responsibility and take effective corrective measures. To explore effective ways to balance economic growth and carbon emission reduction, China’s National Development and Reform Commission (NDRC) identified 74 cities as low-carbon city pilots (LCCP) at the end of 2012 and the beginning of 2017. LCCP aims to implement a low-carbon economy in cities to promote ecological civilization and green development and tackle climate change. In order to foster an emerging economic growth point characterized by low energy consumption, low pollution and low carbon emissions, the first batch of carbon emission trading (CET) pilots were launched in China in 2013. The expansion of the carbon emission trading market has promoted new industrial opportunities. Therefore, it is believed that the Environmental Protection Interview, LCCP and CET have had a certain effect on EEQ, indicating that the effect of MERCOCL on EEQ may be overestimated. To accurately test the promotion effect, dummy variables of Environmental Protection Interview (*talk*), LCCP (*low*) and CET (*cet*) were added to the benchmark model. Suppose the effect of MERCOCL on EEQ was no longer significant after dummy variables were added. In that case, it indicates the effect did not exist and that the study results were not robust. In contrast, the policy effect was significant, but the coefficient decreased, indicating that the research results were overestimated. However, this phenomenon did not affect the research conclusion and can still verify the relative robustness of the results.

In Table 8, after *talk*, *low* and *cet* were added to Model (1) and the regression coefficient and significance of *dt* remained almost unchanged. This indicates that the conclusion that MERCOCL can improve EEQ was relatively stable.

### 5.6. Heterogeneity Analysis of Regional Characteristics

In order to clarify the role of different regional characteristics in the impact of MERCOCL on EEQ, a regional heterogeneity analysis was conducted. MERCOCL improved EEQ mainly by enhancing land market competition, breaking the dual urban-rural land system, optimizing the regional industrial structure and changing the extensive economic development mode. In the long term, local governments have monopolized the primary land market and introduced industrial land supply at low prices. The purpose was to gain a competitive economic advantage and compensate for the lack of regional financial resources [33,48]. There were also significant differences in surface vegetation types, temperature and humidity, which can significantly impact EEQ in southern and northern China [23]. The improvement effect of MERCOCL on EEQ may vary due to the influence of regional economic development and latitude (southern or northern China). Therefore, a heterogeneity analysis was conducted from the perspective of regional differences in economic development levels and latitudes. This paper divided the research sample into high-level and low-level economic development areas. Based on the practice of existing scholars [46], this paper took the Qinling Mountains-Huaihe River as the dividing line between high and low latitudes, thus dividing the research samples into southern and northern areas. The heterogeneity analysis results are shown in Table 9. The coefficients *dt* were significantly positive in counties with a higher economic development level and in the southern region. In comparison, the coefficients in counties with a lower economic development level and in the northern region were insignificant. The implementation of the MERCOCL policy in a region with a higher level of economic development will significantly increase the EEQ level by 0.337. The implementation of the MERCOCL policy in the southern region will significantly increase the EEQ level by 0.385. This conclusion is similar to [4], i.e., land marketization in economically developed areas can better reduce local environmental pollution [4]. In counties with a higher economic development level and in the southern region, MERCOCL had a more significant improvement effect on EEQ. The regions with a higher economic development level and the southern region had a higher degree of industrial structure optimization and a more concentrated population. Thus, the environmental optimization effect of industrial structure optimization [49] and the energy sharing and saving effect of population aggregation [37] can be better promoted. In addition, Sun (2020) summarized and compared MERCOCL implementation cases in various regions and found that the overall effect of the reform in the southern region was better than that in the northern region [50]. This facilitates the contribution of MERCOCL to pollution control.

## 6. Conclusions

As an important part of China’s rural land marketization reform process, the MERCOCL policy significantly improved EEQ. However, the effects have been rarely studied. Therefore, a universal assessment model of China’s regional EEQ was built based on the Landsat 5/8 and the national EI provided by the Ministry of Ecology and Environment at the national district and county scale. A total of 229 Chinese counties (32 pilot counties and other counties in the pilot cities) from 2011 to 2018 were taken as the research objects. This paper empirically studied the evolution process, driving mechanism and spatial heterogeneity of EEQ from the perspective of MERCOCL, using the DID, the PSM-DID model and the intermediary effect model. The main conclusions are as follows:

(1) China’s EEQ presented a spatial distribution pattern of “high in the south, low in the north, high in the east and low in the west”. In areas with densely distributed forests, the EEQ was generally higher.
(2) MERCOCL had a significant improvement effect on EEQ, passing robustness tests. When a county implemented the MERCOCL policy, its eco-environmental quality index increased by 0.342. However, the improvement effect of the MERCOCL policy on EEQ did not appear until the second year after the implementation of the policy. It took some time for MERCOCL to play its ecological value.
(3) Regarding the mechanism, MERCOCL reduced the phenomenon of governments attracting backward industries and the EEQ degradation caused by excessive industrialization. MERCOCL was conducive to attracting population into cities, giving full play to the agglomeration effect of increased population density and promoting the improvement of EEQ.
(4) From the perspective of spatial heterogeneity, the regions with a higher economic development level and the southern region had a higher degree of industrial structure optimization and a more concentrated population. MERCOCL had a more significant improvement effect on EEQ in regions with lower economic development levels and lower latitudes (southern China). The conclusions indicate that the implementation of the MERCOCL policy in a region with a higher level of economic development will significantly increase the EEQ level by 0.337. The implementation of the MERCOCL policy in the southern region will significantly increase the EEQ level by 0.385.

The MERCOCL pilot is a new important measure of China’s land system reform, which has strongly promoted the market-oriented reform of China’s rural land. As an important part of China’s rural land market-oriented reform process, this study discussed the impact of MERCOCL reform on EEQ and the spatio-temporal evolution characteristics of EEQ at the district and county level in China in depth. This will deepen the understanding of MERCOCL’s ecological effects and provide scientific data support for scientists, policymakers, companies, investors and civil society to mitigate the eco-environmental risks from China’s future rural land marketization. In addition, this study also analyzed the driving effect of MERCOCL on EEQ from the perspectives of industrial structure optimization, population aggregation and government intervention. This will provide an effective path reference for the MERCOCL policy to achieve ecological and environmental benefits.

However, this study also has some limitations. Firstly, although the EEQ data produced in this study have good accuracy in China, the spatial resolution of CHEQ data (only 1000 m) is currently limited by factors such as the availability of remote sensing data and the calculation speed. The resolution is insufficient for counties with small areas. This may bring some uncertainties to the results. In the future, more in-depth research will be conducted to improve the spatial resolution of CHEQ data. Attempts will be made to increase the spatial resolution of CHEQ to 30 m in the next stage. Secondly, only the samples up to 2018 are used due to data availability. Thus, it is difficult to investigate the long-term effect of the MERCOCL policy. In the subsequent research, samples over a longer period will be selected to investigate the long-term impact of the MERCOCL policy on EEQ. Finally, this study only examines the impact of the MERCOCL policy on local EEQ without considering the spillover effect of the MERCOCL policy, i.e., the impact of MERCOCL on EEQ in surrounding areas. Thus, the spillover effect of the MERCOCL policy will also be focused on.

Finally, local governments should actively promote a successful experience of MERCOCL to the whole country to effectively solve the problems of insufficient land supply in the urban construction expansion and status inequality in the transformation of agricultural land into non-agricultural land. It is also necessary to actively guide the effective implementation of MERCOCL, form a reasonable land transfer price and reduce the ecological environment damage due to irrational land use.

## Figures and Tables

**Table 1:** Detailed description of remote sensing data.

Data	Format	Spatial Resolution	Temporal Resolution	Source
Landsat 5	TIFF	30 m	16-day	USGS
Landsat 7	TIFF	30 m	16-day	USGS
Landsat 8	TIFF	30 m	16-day	USGS
MCD12Q1	HDF	500 m	Annual	NASA

NASA: National Aeronautics and Space Administration (https://www.nasa.gov/, accessed on 2 June 2022). USGS: United States Geological Survey (https://www.usgs.gov/, accessed on 2 June 2022).

**Table 2:** Descriptive statistics of variables.

Variables	Indicator Meaning	Observation	Mean	S.D.	Minimum	Maximum
EEQ	Ecological environment quality (-)	1832	54.324	12.196	24.896	89.521
ey	Economic growth (10,000 $)	1832	1.105	1.061	0	6.3
s	Industrial structure (%)	1832	44.932	15.426	4.67	92.85
den	Population density (-)	1832	5.740	0.923	2.792	8.657
pop	Population agglomeration (%)	1832	30.587	19.024	8.18	99.67
str	Industrial structure optimization (%)	1832	36.819	10.816	10.3	75.26
gov	Government intervention (%)	1832	24.132	16.699	2.26	105.58
temp	Temperature (°C)	1832	14.39	4.54	3.01	24.76
rain	Rainfall (mm)	1832	990.34	460.44	197.17	2516.57

**Table 3:** Results analysis of MERCOCL on the effect of EEQ.

Variables	(1)	(2)	(3)	(4)	(5)	(6)	(7)
	EEQ	EEQ	EEQ	EEQ	EEQ	EEQ	EEQ
dt	0.342 **	0.392 **	0.389 **	0.454 ***	0.459 ***	0.400 **	0.444 **
	(0.171)	(0.167)	(0.168)	(0.169)	(0.169)	(0.173)	(0.180)
ey		1.169 ***	2.518 ***	2.047 ***	2.042 ***	1.786 ***	1.862 ***
		(0.245)	(0.577)	(0.603)	(0.610)	(0.603)	(0.615)
ey^2^			−0.284 ***	−0.224 **	−0.229 **	−0.202 *	−0.210 *
			(0.109)	(0.109)	(0.111)	(0.108)	(0.111)
s				0.022 *	0.022 *	0.026 **	0.022 *
				(0.013)	(0.013)	(0.013)	(0.013)
den					−0.696	−0.603	−0.536
					(0.851)	(0.900)	(0.885)
rain						0.0008 ***	0.0008 ***
						(0.0001)	(0.0001)
temp							−0.114
							(0.085)
Intercept term	54.297 ***	53.001 ***	52.177 ***	51.575 ***	55.591 ***	54.295 ***	55.655 ***
	(0.014)	(0.272)	(0.420)	(0.582)	(5.030)	(5.342)	(5.434)
Year fixed	YES	YES	YES	YES	YES	YES	YES
County fixed	YES	YES	YES	YES	YES	YES	YES
Observation	1832	1832	1832	1832	1832	1832	1832
*R* ^2^	0.003	0.017	0.023	0.027	0.027	0.034	0.035

Note: ***, ** and * represent the significance levels of 1%, 5%, and 10%, respectively.

**Table 4:** Applicability test of the PSM-DID method (common support hypothesis).

Variables	Mean of the Treatment Group	Mean of the Control Group	Difference	*t* Value	*p* Value
ey	1.325	1.178	0.147	1.22	0.222
ey^2^	2.883	2.322	0.561	0.99	0.324
s	46.954	47.445	−0.491	−0.30	0.765
den	5.953	5.903	0.050	0.50	0.621
temp	13.836	13.903	−0.067	−0.12	0.902

**Table 5:** Results analysis of the impact of MERCOCL on EEQ using PSM-DID.

Variables	(1)	(2)	(3)	(4)	(5)	(6)	(7)
	EEQ	EEQ	EEQ	EEQ	EEQ	EEQ	EEQ
dt	0.397 **	0.419 **	0.403 **	0.498 ***	0.510 ***	0.461 ***	0.490 ***
	(0.169)	(0.167)	(0.169)	(0.171)	(0.171)	(0.175)	(0.182)
ey		0.905 ***	2.534 ***	1.800 ***	1.787 ***	1.651 ***	1.742 ***
		(0.227)	(0.541)	(0.575)	(0.578)	(0.590)	(0.595)
ey^2^			−0.365 ***	−0.263 ***	−0.269 ***	−0.253 ***	−0.266 ***
			(0.096)	(0.092)	(0.090)	(0.091)	(0.092)
s				0.030 **	0.030 **	0.032 **	0.029 **
				(0.014)	(0.014)	(0.014)	(0.015)
den					−1.324	−1.353	−1.248
					(1.083)	(1.110)	(1.114)
rain						0.0005 ***	0.0006 ***
						(0.0002)	(0.0002)
temp							−0.090
							(0.091)
Intercept term	53.555 ***	52.580 ***	51.585 ***	50.768 ***	58.502 ***	58.109 ***	58.846 ***
	(0.015)	(0.246)	(0.406)	(0.601)	(6.465)	(6.614)	(6.707)
Year fixed	YES	YES	YES	YES	YES	YES	YES
County fixed	YES	YES	YES	YES	YES	YES	YES
Observation	1646	1646	1646	1646	1646	1646	1646
*R* ^2^	0.004	0.012	0.021	0.027	0.029	0.032	0.033

Note: Control variables are gradually added to Columns (1)–(7) in the same order as Table 3. ***, ** represent the significance levels of 1% and 5%, respectively.

**Table 6:** Driving mechanism test of MERCOCL on EEQ.

Variables	Benchmark Regression	Structure Optimization		Government Intervention		Population Agglomeration	
	(1)	(2)	(3)	(4)	(5)	(6)	(7)
	EEQ	str	EEQ	gov	EEQ	pop	EEQ
dt	0.444 **	1.279 *	0.439 **	−0.829	0.443 **	4.794 ***	0.307 *
	(0.180)	(0.721)	(0.180)	(0.697)	(0.180)	(1.674)	(0.182)
str			0.003 **				
			(0.0015)				
gov					−0.001		
					(0.008)		
pop							0.029 ***
							(0.007)
Intercept term	55.655 ***	25.544	55.568 ***	40.425 ***	55.609 ***	−132.54 **	59.436 ***
	(5.435)	(20.997)	(10.26)	(14.343)	(5.387)	(65.431)	(4.347)
Control variable	YES	YES	YES	YES	YES	YES	YES
Year fixed	YES	YES	YES	YES	YES	YES	YES
County fixed	YES	YES	YES	YES	YES	YES	YES
Observations	1832	1832	1832	1832	1832	1832	1832
*R* ^2^	0.035	0.093	0.036	0.095	0.035	0.163	0.067

Note: ***, ** and * represent the significance levels of 1%, 5%, and 10%, respectively.

**Table 7:** Time sensitivity test.

Variables	2014–2016	2013–2017	2012–2018
	EEQ	EEQ	EEQ
dt	0.273 *	0.298 **	0.493 ***
	(0.145)	(0.150)	(0.182)
Intercept term	60.239 ***	54.299 ***	56.339 ***
	(5.810)	(6.306)	(6.244)
Control Variable	YES	YES	YES
Year fixed	YES	YES	YES
County fixed	YES	YES	YES
Observations	687	1145	1603
*R* ^2^	0.043	0.078	0.039

Note: ***, ** and * represent the significance levels of 1%, 5%, and 10%, respectively.

**Table 8:** Policy interference test.

Variables	(1)	(2)	(3)	(4)	(5)
	EEQ	EEQ	EEQ	EEQ	EEQ
dt	0.444 **	0.444 **	0.437 **	0.427 **	0.424 **
	(0.180)	(0.181)	(0.180)	(0.184)	(0.185)
talk		−0.295			−0.287
		(0.196)			(0.194)
low			0.113		0.041
			(0.133)		(0.135)
cet				0.555 ***	0.548 ***
				(0.145)	(0.147)
Intercept term	55.655 ***	54.957 ***	55.859 ***	57.421 ***	56.793 ***
	(5.435)	(5.573)	(5.470)	(5.362)	(5.523)
Control variable	YES	YES	YES	YES	YES
Year fixed	YES	YES	YES	YES	YES
County fixed	YES	YES	YES	YES	YES
Observations	1832	1832	1832	1832	1832
*R* ^2^	0.035	0.038	0.036	0.041	0.043

Notice: Column (1) shows the model that only includes the MERCOCL; Column (2) shows the model that includes talk (Environmental Protection Interview); Column (3) adds low (low-carbon city pilots); Column (4) adds cet (carbon emission trading); Column (1) shows the model that includes all policies. ***, ** represent the significance levels of 1% and 5%, respectively.

**Table 9:** Heterogeneity analysis of regional characteristics.

Variables	Higher Economic Development Level	Lower Economic Development Level	Southern Region	Northern Region
	EEQ	EEQ	EEQ	EEQ
*dt*	0.337 **	0.390	0.385 **	0.436
	(0.168)	(0.321)	(0.191)	(0.304)
Intercept term	51.623 ***	55.921 ***	49.052 ***	62.749 ***
	(3.603)	(11.608)	(4.141)	(9.722)
Control variable	YES	YES	YES	YES
Year fixed	YES	YES	YES	YES
County fixed	YES	YES	YES	YES
7	616	1216	1016	816
*R* ^2^	0.070	0.071	0.077	0.031

Note: ***, ** represent the significance levels of 1% and 5%, respectively.

## Data Availability

Data sharing is not applicable to this article.
